# Long-term risk of cardiovascular mortality according to age group and blood pressure categories of the latest guideline

**DOI:** 10.1038/s41440-025-02151-w

**Published:** 2025-02-20

**Authors:** Michihiro Satoh, Takayoshi Ohkubo, Katsuyuki Miura, Akiko Harada, Anna Tsutsui, Atsushi Hozawa, Yuji Shimizu, Shizukiyo Ishikawa, Yoshihiro Kokubo, Tomonori Okamura, Yoshitaka Murakami, on behalf of the Evidence for Cardiovascular Prevention from Observational Cohorts in Japan (EPOCH–JAPAN) Research Group

**Affiliations:** 1 Division of Public Health, Hygiene and Epidemiology, Faculty of Medicine, Tohoku Medical and Pharmaceutical University, Sendai, Japan; 2 Department of Preventive Medicine and Epidemiology, Tohoku Medical Megabank Organization, Tohoku University, Sendai, Japan; 3 Department of Pharmacy, Tohoku Medical and Pharmaceutical University Hospital, Sendai, Japan; 4 Department of Hygiene and Public Health, Teikyo University School of Medicine, Tokyo, Japan; 5 Tohoku Institute for Management of Blood Pressure, Sendai, Japan; 6 NCD Epidemiology Research Center, Shiga University of Medical Science, Otsu, Japan; 7 Department of Medical Statistics, Toho University School of Medicine, Tokyo, Japan; 8 Division of Epidemiology, Graduate School of Medicine, Tohoku University, Sendai, Japan; 9 Department of General Medicine, Nagasaki University Graduate School of Biomedical Sciences, Nagasaki, Japan; 10 Epidemiology Section, Division of Public Health, Osaka Institute of Public Health, Osaka, Japan; 11 Center for Information, Jichi Medical University, Tochigi, Japan; 12 Department of Preventive Cardiology, National Cerebral and Cardiovascular Center, Suita, Japan; 13 Department of Preventive Medicine and Public Health, Keio University School of Medicine, Tokyo, Japan

**Keywords:** Blood pressure, Meta-analysis, Epidemiology, Cardiovascular diseases, Population

## Abstract

This study examined the association between the latest blood pressure (BP) classification and cardiovascular disease (CVD) mortality risk, using data from 70,570 individuals across 10 Japanese cohorts. Participants were stratified by age (40–64 and 65–89 years) and antihypertensive treatment use. BP was classified according to the 2019 Japanese Society of Hypertension Guidelines. During a follow-up period of approximately 10 years, 2304 CVD deaths occurred. Cox models demonstrated that CVD mortality risk increased stepwise with the BP category, with this association being especially pronounced in patients aged 40–64 years, where the Grade I hypertension group showed the highest population-attributable fraction (PAF). When the treated participants were included in the hypertension group, the overall PAF for CVD mortality was 41.1%. Similar patterns were observed for CVD subtype mortality risk, with hypertension showing particularly high PAFs for intracerebral hemorrhage. These findings highlight the importance of early-stage prevention and management of hypertension.

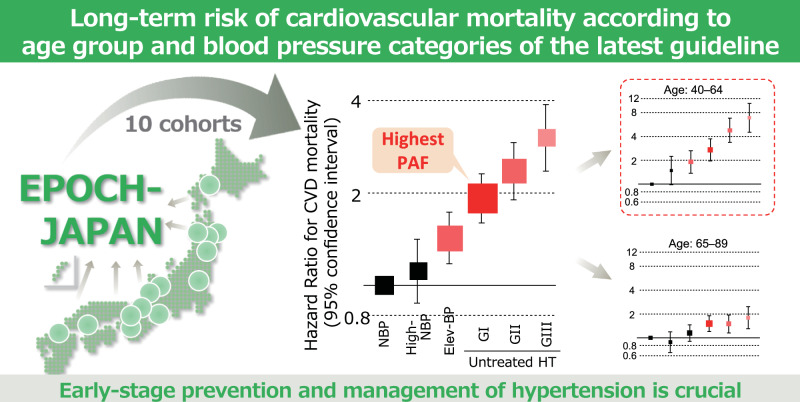

## Introduction

Hypertension remains a strong risk factor for cardiovascular disease (CVD) and contributes substantially to global morbidity and mortality. Previous research conducted by the Evidence for Cardiovascular Prevention from Observational Cohorts in Japan (EPOCH-JAPAN) Research Group demonstrated a clear association between blood pressure (BP) and CVD mortality risk, with a particularly notable effect observed among younger populations [1]. However, the previous analysis included participants who received antihypertensive treatment.

In 2019, the Japanese Society of Hypertension (JSH) updated its guidelines and revised the threshold values for BP classification [2]. These BP threshold values align with those used in the United States guidelines, although the names of the BP categories differ [3]. Therefore, reassessing the association between BP categories and CVD mortality risk based on these newly defined BP categories could provide valuable insights into contemporary clinical practice. Such reassessment would help inform decision-making by healthcare providers and policymakers aimed at reducing CVD burden in Japan.

This study aimed to quantify the association between the latest BP classifications and CVD mortality risk using data from the EPOCH-JAPAN, particularly for untreated individuals.

Point of view

- **Clinical relevance**
  The risk of cardiovascular mortality increased stepwise with the latest blood pressure category. This association was particularly pronounced in participants aged 40–64 years, among whom the Grade I hypertension group demonstrated the highest population-attributable fraction.
- **Future direction**
  Further research utilizing contemporary large-scale data, including treatment status during follow-up, out-of-office blood pressure measurements, and lifestyle factors, is required to reflect more recent and detailed circumstances.
- **Consideration for the Asian population**
  In Asian populations, who traditionally experience higher stroke incidence rates, the prevention of hypertension substantially reduces the cardiovascular disease burden, especially among younger individuals.

## Methods

The full version of the methods is provided in the Supplementary Data section.

### Study design and population

The EPOCH-JAPAN study is a meta-analysis of individual participant data from Japanese cohorts [4, 5]. The study protocol was approved by the Institutional Review Board. Using inclusion criteria similar to those in a previous report [1], our final analysis included 70,570 individuals from 10 cohort studies (the participant selection flow is shown in Supplementary Fig. 1). The baseline years ranged from 1980–2002 [4, 5].

### BP measurement and covariates

Routine health examinations were performed during the baseline survey, including BP measurements, blood tests, questionnaires, and body measurements. BP was measured using a mercury sphygmomanometer with participants in a seated position, except in the Ohasama and Iwate-Kenpoku cohort studies, where an automated device was used [4–6]. Other details are provided elsewhere [1, 7, 8]. Based on the JSH 2019 guidelines [2], we classified untreated participants into the following categories based on systolic/diastolic BP: <120/<80 mmHg as “Normal BP,” 120–129/<80 mmHg as “High-normal BP,” 130–139/80–89 mmHg as “Elevated BP,” 140–159/90–99 mmHg as grade (G) I hypertension, 160–179/100–109 mmHg as GII hypertension, and ≥180/≥110 mmHg as GIII hypertension. We used the hypertension category, defined as BP ≥140/≥90 mmHg or under antihypertensive treatment.

### Outcomes

We defined the following outcomes using the 9^th^ and 10^th^ International Classification of Diseases: total CVD (390–459; I00–I99), total stroke (430–438; I60–I69), ischemic stroke (433, 434, or 437.8; I63 or I69.3), intracerebral hemorrhage (431–432; I61 or I69.1), coronary heart disease (410–414; I20–I25), and heart failure (428 or I50) [4, 9].

### Statistical analysis

We estimated the hazard ratios (HRs) for total CVD mortality by using Cox proportional hazards models. A leave-one-out analysis was performed to determine whether any specific cohort significantly influenced the results. The reference and nonhypertensive groups had the lowest BP values. We categorized the participants into two age groups at baseline: “middle-aged (aged 40–64 years) and older adults (65–89 years). Stratified Cox models were used to account for cohort heterogeneity. The population attributable fraction (PAF) was calculated using the following formula:

$${{{\rm{PAF}}}}={{{\rm{pd}}}}({{{\rm{RR}}}}-1)/{{{\rm{RR}}}}$$

In this equation, “pd” represents the proportion of fatalities among those exposed within a particular BP group. “RR” denotes the corresponding fully-adjusted HR [1].

All statistical analyses were performed using SAS version 9.13 (SAS Institute, Cary, NC, USA). All *P* values for statistical tests were two-tailed, and *P*-values < 0.05 were regarded as statistically significant.

## Results

### Baseline characteristics

Of the 70,570 participants (57.1% women; mean age, 59.1 years), 57,656 (81.7%) were untreated. Normal BP was observed in 18,355 (31.8%) participants, high-normal BP in 7526 (13.1%), elevated BP in 15,442 (26.8%), GI hypertension in 11,772 (20.4%), GII hypertension in 3592 (6.2%), and GIII hypertension in 969 (1.7%) participants (Supplementary Table 1). The remaining 12,914 participants (18.3%) were treated for hypertension (characteristics after combining untreated and treated hypertensive participants are represented in Supplementary Table 2).

### BP category and total CVD mortality risk

**Fig. 1:**
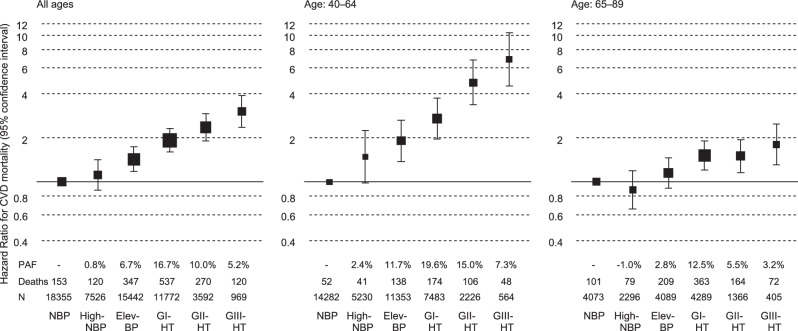
BP categories and total CVD mortality risk stratified by age in untreated participants. Hazard ratios were adjusted for sex, age, total cholesterol, ex-smoking, current smoking, ex-drinking, current drinking, body mass index (<18.5, ≥25 kg/m^2^), and diabetes. The sizes of the marker boxes indicate the number of events in each group. BP blood pressure, CVD cardiovascular disease, NBP normal BP, High-NBP high-normal BP, Elev-BP elevated BP, HT hypertension (*untreated participants with BP ≥140/≥90 mmHg and treated participants)

**Fig. 2:**
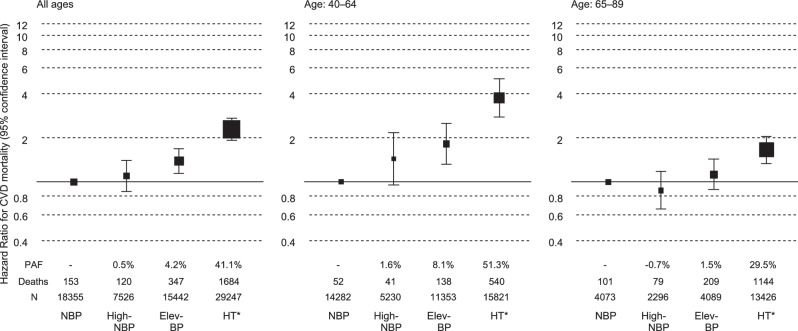
BP categories and total CVD mortality risk stratified by age when including treated participants in the HT group. Hazard ratios were adjusted for sex, age, total cholesterol, ex-smoking, current smoking, ex-drinking, current drinking, body mass index (BMI, <18.5 and ≥25 kg/m^2^), and diabetes. The sizes of the marker boxes indicate the number of events in each group. BP blood pressure, CVD cardiovascular disease, NBP normal BP, High-NBP high-normal BP, Elev-BP elevated BP, HT hypertension (*untreated participants with BP ≥140/≥90 mmHg and treated participants)

A total of 2304 CVD-related deaths occurred during the mean follow-up period of 9.9 years. Among untreated participants, the risk of total CVD mortality increased stepwise with the BP category, particularly in younger populations, while the highest PAF for total CVD mortality was observed in the GI hypertension group among untreated participants (Fig. 1 and Supplementary Table 3). When treated participants were included in the hypertension group, the PAF in the hypertension group was 41.1% of the entire population (Fig. 2 and Supplementary Table 4). These results did not change after excluding each cohort (Supplementary Tables 5 and 6): hazard ratios for total CVD of hypertension ranged 2.22–2.44 in the leave-one-out analyses (Supplementary Table 6).

### BP category and mortality risk from CVD subtype

Among untreated participants, BP was significantly associated with mortality risks from total stroke, ischemic stroke, intracerebral hemorrhage, coronary heart disease, and heart failure. The PAFs for CVD subtype mortality were most pronounced in the GI hypertension group (Supplementary Fig. 2 and Supplementary Table 7). When treated participants were included in the hypertension group, the PAFs reached 29.5% or more across the entire study population, with the highest PAF (57.1%) observed for intracerebral hemorrhage mortality (Supplementary Fig. 3 and Supplementary Table 8).

## Discussion

We analyzed the CVD risk based on the latest BP classification in the JSH guidelines. Furthermore, we defined the BP categories by focusing on untreated individuals to address the key limitations of our previous study [1].

Similar to a previous study [1], an association between hypertension and the total CVD mortality risk was observed in the entire study population and became clearer in the younger population. Considering its lifetime risk [8, 10, 11], the prevention of hypertension at a younger age is crucial. A previous study of untreated early to middle-aged adults (mean age: 41.3 years) demonstrated that the BP categories that we adopted were strongly associated with CVD event risk, with the elevated BP group showing the highest PAF of 17.8% [12]. The baseline of the previous study was 2010–2011, whereas that of our study was around the 1990s. With advances in hypertension management and improved treatment rates since the 1990s, BP levels in untreated individuals in Japan have likely decreased [2]. Consequently, populations with high PAFs for CVD may have shifted toward lower BP levels in recent years. Regarding CVD subtypes, the PAF of high BP was markedly higher for intracerebral hemorrhage than for the other subtypes. This may be because high BP can directly cause intracerebral hemorrhage with relatively less influence from other CVD risk factors [13].

The attenuated association between BP and total CVD mortality risk in participants aged 65–89 years may be attributed to the inclusion of individuals with decreased BP due to adverse health conditions in the non-hypertensive older adult population. Another possible explanation is survival bias, because the older age group likely excluded individuals who had died from CVD due to severe hypertension before reaching that age. Nevertheless, the total CVD mortality risk of hypertension remained substantial, with a PAF of 29.5% in participants aged 65–89 years. The contribution of hypertension to the total CVD burden remains considerable, even in the older adult population, given its high absolute risk.

### Perspective of Asia

The present results of elevated CVD risks according to higher BP have significant implications for preventive strategies across Asia, especially for younger populations. Asian populations traditionally have higher stroke rates compared to Western populations [13], with hypertension being a major contributing factor [2]. The findings regarding the high PAF for CVD mortality in hypertensive individuals suggest that the prevention of hypertension could substantially reduce the proportion of CVD in Asian populations.

### Limitations

This study has certain limitations. First, the pooled data did not include the participants’ status during follow-up. Participants with high BP must have been treated with antihypertensive medications. Second, this study was based on office BP measurements obtained on a single occasion. Third, our analysis was limited to the mortality outcomes. The Japan Arteriosclerosis Longitudinal Study (JALS) demonstrated similar associations between BP categories and the incidence of stroke and myocardial infarction, although this previous study did not examine age-stratified analyses [14]. Finally, although salt intake and physical activity levels were potential confounders, the data on these factors were not included in the present database. In particular, the salt intake in Japan during the 1990s, when this study’s baseline survey was performed, was high and could have had a substantial impact.

### Conclusion

In conclusion, this updated analysis of the EPOCH-JAPAN study demonstrated an association between the latest BP classification and the total CVD mortality risk, particularly in younger populations. Furthermore, given the high PAF for total CVD mortality in individuals with hypertension, our findings underscore the critical importance of early prevention and management of hypertension. Complete prevention of hypertension has been estimated to reduce total CVD mortality by approximately 50% and 30% in non-older and older adult populations, respectively.

## Supplementary information

Supplementary Information

## Data Availability

The authors declare that all supporting data are available in the article and online supplementary files.
