# Supplementary material for: Long-term risk of cardiovascular mortality according to age group and blood pressure categories of the latest guideline
Source: Hypertens Res. 2025 Feb 20;48(4):1428–33. doi: 10.1038/s41440-025-02151-w (PMC11972951; doi:10.1038/s41440-025-02151-w)
Supplement: Supplementary file 1 — Supplementary Information [file 41440_2025_2151_MOESM1_ESM.pdf]

Supplementary Information

Supplemental to:

Long-term risk of cardiovascular disease according to age group and blood pressure categories of the latest guideline

Methods (Full version)

Study Design and Populations

The Evidence for Cardiovascular Prevention from Observational Cohorts in Japan (EPOCH-JAPAN) study is a meta-analysis of individual participant data from Japanese cohorts [4,16]. The EPOCH-JAPAN study draws on datasets comprised of individual participant data from longitudinal observational studies conducted across Japan. Each study contributing to the EPOCH-JAPAN project received ethical approval from its respective institutional review boards. The EPOCH-JAPAN study received ethical approval from two key institutions: the Institutional Review Board of Shiga University of Medical Science (23-125-1), the Ethics Committee of the Keio University School of Medicine (20110192), and the Ethics Committee of Toho University Faculty of Medicine (A22090_A20042). The former serves as the data management center, while the latter two are affiliated with the principal investigator of the EPOCH-JAPAN study. Using inclusion criteria similar to those in a previous report [1], our final analysis included 70,570 individuals from 10 cohort studies (the participant selection flow is shown in **Supplementary Figure 1**). The baseline years ranged from 1980–2002 [4,5].

BP measurement and covariates

During the baseline survey, we performed routine health examinations, including blood pressure (BP) measurements, blood tests, questionnaires, and body measurements. BP was measured using a mercury sphygmomanometer with participants in a seated position, except in the Ohasama and Iwate-Kenpoku cohort studies, where an automated device was used [4-6]. Other details are provided elsewhere [1,7,8].

Based on the JSH 2019 guidelines [2], we classified untreated participants into the following categories based on systolic/diastolic BP: < 120/< 80 mmHg as “Normal BP,” 120–129/< 80 mmHg as “High-normal BP,” 130–139/80–89 mmHg as “Elevated BP,” 140–159/90–99 mmHg as grade (G) I hypertension, 160–179/100–109 mmHg as GII hypertension, and ≥ 180/≥ 110 mmHg as GIII hypertension. We used the hypertension category, defined as BP ≥ 140/≥ 90 mmHg or under antihypertensive treatment. In cases where an individual's systolic and diastolic BP corresponded to different categories, we assigned the participant to a higher BP category. Diabetes was defined as fasting (≥ 126 mg/dL) or non-fasting (≥ 200 mg/dL) plasma

glucose levels and/or the current use of antidiabetic medications. Body mass index (BMI) was calculated as weight (kg) divided by height in meters squared (m^2). Participants were classified as current-, quit- (ex-), and non-smokers, and drinking as current-, quit- (ex-), and non-drinkers.

Outcomes

In accordance with the Family Registration Law in Japan, all death certificates are forwarded to the Ministry of Health, Labor, and Welfare via the public health center in the area of residence. Registration of deaths is required by law. Other sources used in a few studies include autopsy reports, medical records, health examinations, and questionnaires [1,7,8]. We defined the following outcomes using the 9th and 10th International Classification of Diseases: total CVD (390–459; I00–I99), total stroke (430–438; I60–I69), ischemic stroke (433, 434, or 437.8; I63 or I69.3), intracerebral hemorrhage (431–432; I61 or I69.1), coronary heart disease (410–414; I20–I25), and heart failure (428 or I50) [9,10].

Statistical Analysis

We estimated the hazard ratios (HRs) of total CVD mortality using Cox proportional hazards models. The reference group was the lowest BP, or the non-hypertensive group. A leave-one-out analysis was performed to determine whether any specific cohort significantly influenced the results. We categorized participants into two age groups at baseline: "middle-aged" represented those 40–64 years old, and "elderly" represented those 65–89 years old. Stratified Cox models were used to account for cohort heterogeneity. Two statistical models were used to adjust for the potential confounders. First, we adjusted the results for age and sex (sex- and age-adjusted model) and then further adjusted for total cholesterol, ex-smoking, current smoking, ex-drinking, current drinking, body mass index categorized as < 18.5 and ≥ 25 kg/m^2, and diabetes (fully adjusted model).

The population attributable fraction (PAF) was derived using the formula: PAF = pd(RR-1)/RR. In this equation, pd represents the proportion of fatalities among those exposed within a particular BP group. RR denotes the fully-adjusted HR in the current study [1].

All the statistical analyses were performed using SAS version 9.13 (SAS Institute, Cary, NC, USA). All P values for statistical tests were two-tailed, and $P < 0.05$ was regarded as statistically significant.

EPOCH-JAPAN Research Group: The Evidence for Cardiovascular Prevention from Observational Cohorts in Japan (EPOCH–JAPAN) Research Group is composed of the following investigators.
Co-chairpersons: Hirotsugu Ueshima (Shiga University of Medical Science), Tomonori Okamura (Keio University School of Medicine), Yoshitaka Murakami (Toho University).
Executive committee: Hirotsugu Ueshima (Shiga University of Medical Science), Yutaka Kiyohara (Hisayama Research Institute for Lifestyle Diseases), Toshiharu Ninomiya (Kyushu University Graduate School of Medicine), Yutaka Imai (Tohoku Institute for Management of Blood Pressure), Takayoshi Ohkubo (Teikyo University School of Medicine), Hiroyasu Iso (The National Center for Global Health and Medicine), Isao Muraki (Osaka University Graduate School of Medicine), Kazumasa Yamagishi (Juntendo University Graduate School of Medicine, University of Tsukuba Institute of Medicine), Akiko Tamakoshi (Hokkaido University Faculty of Medicine), Yoshihiro Miyamoto (National Cerebral and Cardiovascular Center), Yoshihiro Kokubo (National Cerebral and Cardiovascular Center), Katsuyuki Miura (Shiga University of Medical Science), Sachiko Tanaka (Kyoto University Graduate School of Medicine and Public Health), Akiko Harada (Shiga University of Medical Science), Shigeyuki Saitoh (Sapporo Medical University), Hirofumi Ohnishi (Sapporo Medical University), Ichiro Tsuji (Tohoku University Graduate School of Medicine), Atsushi Hozawa (Tohoku University), Hideaki Nakagawa (Kanazawa Medical University), Masaru Sakurai (Kanazawa Medical University), Yoshimi Tatsukawa (Radiation Effects Research Foundation), Kiyomi Sakata (Iwate Medical University), Kozo Tanno (Iwate Medical University), Akihiko Kitamura (Yao City Public Health Center), Masahiko Kiyama (Institute of Preventive Medicine, Inc.), Yuji Shimizu (Osaka Institute of Public Health), Akira Okayama (Research Institute of Strategy for Prevention), Shizukiyo Ishikawa (Jichi Medical University), Hiroshi Yatsuya (Nagoya University Graduate School of Medicine), Takeo Nakayama (Kyoto University School of Public Health), Fujiko Irie (Ibaraki Prefecture), and Toshimi Sairenchi (Dokkyo Medical University).

Supplementary Table 1. Characteristics in untreated participants

Age group<br>Characteristic	NBP	High-NBP	Elev-BP	GI-HT	GII-HT	GIII-HT
All ages						
N	18,355	7,526	15,442	11,772	3,592	969
Women, %	64.7	58.8	51.0	51.2	48.4	46.7
Age, year	55.6	57.9	57.3	60.1	61.1	62.1
Body mass index, kg/m^2	22.5	23.1	23.4	23.8	24.0	24.0
Current smoker, %	20.9	21.9	24.7	24.2	27.6	33.7
Ex-smoker, %	9.7	11.3	13.9	14.7	15.3	12.2
Current drinker, %	39.0	40.0	47.7	45.7	48.0	52.6
Ex-drinker, %	3.0	2.8	2.9	3.1	3.0	2.4
Diabetes, %	2.5	4.2	4.2	5.2	5.9	4.4
Systolic BP, mmHg	108.1	123.8	129.8	145.2	162.4	185.3
Diastolic BP, mmHg	66.5	72.0	80.3	86.0	94.2	103.6
Total cholesterol, mmol/L	5.1	5.2	5.3	5.3	5.3	5.3
40–64 years						
N	14,282	5,230	11,353	7,483	2,226	564
Women, %	65.7	58.5	50.2	49.2	45.6	40.6
Age, year	51.4	52.2	52.5	53.8	54.5	54.3
Body mass index, kg/m^2	22.5	23.2	23.5	24.0	24.3	24.5
Current smoker, %	22.2	24.7	26.7	27.4	30.8	39.4
Ex-smoker, %	8.5	9.7	12.9	13.8	14.2	12.9
Current drinker, %	41.9	44.3	51.4	51.4	53.9	62.1
Ex-drinker, %	2.3	1.8	2.0	2.0	2.2	1.1
Diabetes, %	2.0	3.3	3.8	4.8	5.7	4.8
Systolic BP, mmHg	107.9	123.6	129.1	144.1	160.7	182.5
Diastolic BP, mmHg	66.7	72.4	81.0	87.7	96.7	107.6
Total cholesterol, mmol/L	5.1	5.2	5.3	5.3	5.3	5.3
65–89 years						
N	4,073	2,296	4,089	4,289	1,366	405
Women, %	61.3	59.5	53.2	54.6	53.0	55.3
Age, year	70.2	70.8	70.5	71.1	72.0	72.8
Body mass index, kg/m^2	22.4	23.0	23.2	23.4	23.4	23.3
Current smoker, %	16.3	15.7	19.2	18.5	22.5	25.9
Ex-smoker, %	13.8	14.9	16.9	16.3	17.1	11.1
Current drinker, %	28.8	30.1	37.5	35.7	38.5	39.5
Ex-drinker, %	5.6	5.1	5.4	5.1	4.3	4.2
Diabetes, %	4.3	6.1	5.2	5.9	6.3	4.0
Systolic BP, mmHg	108.7	124.2	131.7	147.1	165.2	189.2
Diastolic BP, mmHg	65.7	71.1	78.6	83.1	90.2	98.0
Total cholesterol, mmol/L	5.1	5.2	5.2	5.2	5.2	5.2

BP, blood pressure; NBP, normal BP; High-NBP, high-normal BP; Elev-BP, elevated BP; GI/GII/GIII-HT, grade I/II/III hypertension

Supplementary Table 2. Characteristics when including treated participants in the HT group

Age group<br>Characteristic	NBP	High-NBP	Elev-BP	HT*
All ages				
N	18,355	7,526	15,442	29,247
Women, %	64.7	58.8	51.0	55.2
Age, year	55.6	57.9	57.3	62.6
Body mass index, kg/m^2	22.5	23.1	23.4	24.2
Current smoker, %	20.9	21.9	24.7	21.2
Ex-smoker, %	9.7	11.3	13.9	14.7
Current drinker, %	39.0	40.0	47.7	42.8
Ex-drinker, %	3.0	2.8	2.9	3.6
Diabetes, %	2.5	4.2	4.2	7.6
Systolic BP, mmHg	108.1	123.8	129.8	147.5
Diastolic BP, mmHg	66.5	72.0	80.3	86.3
Total cholesterol, mmol/L	5.1	5.2	5.3	5.3
40–64 years				
N	14,282	5,230	11,353	15,821
Women, %	65.7	58.5	50.2	52.7
Age, year	51.4	52.2	52.5	55.1
Body mass index, kg/m^2	22.5	23.2	23.5	24.4
Current smoker, %	22.2	24.7	26.7	25.6
Ex-smoker, %	8.5	9.7	12.9	13.5
Current drinker, %	41.9	44.3	51.4	49.5
Ex-drinker, %	2.3	1.8	2.0	2.4
Diabetes, %	2.0	3.3	3.8	6.9
Systolic BP, mmHg	107.9	123.6	129.1	147.4
Diastolic BP, mmHg	66.7	72.4	81.0	89.2
Total cholesterol, mmol/L	5.1	5.2	5.3	5.3
65–89 years				
N	4,073	2,296	4,089	13,426
Women, %	61.3	59.5	53.2	58.2
Age, year	70.2	70.8	70.5	71.5
Body mass index, kg/m^2	22.4	23.0	23.2	24.0
Current smoker, %	16.3	15.7	19.2	16.0
Ex-smoker, %	13.8	14.9	16.9	16.0
Current drinker, %	28.8	30.1	37.5	35.0
Ex-drinker, %	5.6	5.1	5.4	5.1
Diabetes, %	4.3	6.1	5.2	8.4
Systolic BP, mmHg	108.7	124.2	131.7	147.5
Diastolic BP, mmHg	65.7	71.1	78.6	82.8
Total cholesterol, mmol/L	5.1	5.2	5.2	5.2

BP, blood pressure; NBP, normal BP; High-NBP, high-normal BP; Elev-BP, elevated BP; HT, hypertension (*untreated participants with BP ≥ 140/≥ 90 mmHg and treated participants).

Supplementary Table 3. Cardiovascular mortality according to BP categories by age group in untreated participants

Age group<br>Data in each age group	NBP	High-NBP	Elev-BP	GI-HT	GII-HT	GIII-HT
All ages						
N of at risk	18,355	7,526	15,442	11,772	3,592	969
N of cardiovascular death	153	120	347	537	270	120
Person-years	164,078	74,551	173,252	129,565	41,160	11,645
Crude rate [a]	0.9	1.6	2.0	4.1	6.6	10.3
Sex/Age-adjusted HR (95% CI)	1.00 (Ref)	1.11 (0.87–1.41)	1.38 (1.14–1.68)	1.86 (1.55–2.24)	2.26 (1.84–2.77)	2.84 (2.22–3.64)
Fully adjusted HR (95% CI) [b]	1.00 (Ref)	1.12 (0.88–1.42)	1.43 (1.18–1.74)	1.92 (1.60–2.32)	2.36 (1.91–2.90)	3.03 (2.36–3.89)
PAF (%) [c]	-	0.8	6.7	16.7	10.0	5.2
40–64 years						
N of at risk	14,282	5,230	11,353	7,483	2,226	564
N of cardiovascular death	52	41	138	174	106	48
Person-years	139,390	58,718	140,109	94,658	29,189	7,927
Crude rate [a]	0.4	0.7	1.0	1.8	3.6	6.1
Sex/Age-adjusted HR (95% CI)	1.00 (Ref)	1.47 (0.98–2.22)	1.84 (1.33–2.55)	2.58 (1.88–3.55)	4.52 (3.21–6.36)	6.32 (4.21–9.49)
Fully adjusted HR (95% CI) [b]	1.00 (Ref)	1.49 (0.99–2.25)	1.91 (1.38–2.64)	2.71 (1.96–3.74)	4.77 (3.37–6.75)	6.83 (4.52–10.33)
PAF (%) [c]	-	2.4	11.7	19.6	15.0	7.3
65–89 years						
N of at risk	4,073	2,296	4,089	4,289	1,366	405
N of cardiovascular death	101	79	209	363	164	72
Person-years	24,688	15,834	33,143	34,907	11,971	3,718
Crude rate [a]	4.1	5.0	6.3	10.4	13.7	19.4
Sex/Age-adjusted HR (95% CI)	1.00 (Ref)	0.88 (0.65–1.18)	1.11 (0.87–1.42)	1.45 (1.16–1.82)	1.42 (1.10–1.84)	1.68 (1.23–2.30)
Fully adjusted HR (95% CI) [b]	1.00 (Ref)	0.88 (0.66–1.19)	1.15 (0.90–1.47)	1.52 (1.20–1.91)	1.50 (1.16–1.95)	1.80 (1.31–2.47)
PAF (%) [c]	-	-1.0	2.8	12.5	5.5	3.2

[a] A crude rate was expressed as per 1,000 person-years.
[b] Covariates were sex, age, total cholesterol, current-/ex-smoking, current-/ex-drinking, body mass index (< 18.5/≥ 25 kg/m^2), and diabetes.
[c] PAF estimate was based on the HR obtained using the fully adjusted model.
Abbreviations: 95% CI, 95% confidence interval; HR, hazard ratio; BP, blood pressure; PAF, population-attributable fraction; NBP, normal BP; High-NBP, high-normal BP; Elev-BP, elevated BP; GI/GII/GIII-HT, grade I/II/III hypertension

Supplementary Table 4. Cardiovascular mortality according to BP categories by age group when including treated participants in the HT group

Age group<br>Data in each age group	NBP	High-NBP	Elev-BP	HT [d]
All ages				
N of at-risk	18,355	7,526	15,442	29,247
N of cardiovascular death	153	120	347	1,684
Person-years	164,078	74,551	173,252	287,793
Crude rate [a]	0.9	1.6	2.0	5.9
Sex/Age-adjusted HR (95% CI)	1.00 (Ref)	1.10 (0.86–1.39)	1.35 (1.11–1.63)	2.22 (1.88–2.63)
Fully adjusted HR (95% CI) [b]	1.00 (Ref)	1.10 (0.86–1.40)	1.39 (1.15–1.68)	2.29 (1.93–2.72)
PAF (%) [c]	-	0.5	4.2	41.1
40–64 years				
N of at-risk	14,282	5,230	11,353	15,821
N of cardiovascular death	52	41	138	540
Person-years	139,390	58,718	140,109	187,690
Crude rate [a]	0.4	0.7	1.0	2.9
Sex/Age-adjusted HR (95% CI)	1.00 (Ref)	1.45 (0.96–2.18)	1.78 (1.29–2.46)	3.73 (2.79–4.99)
Fully adjusted HR (95% CI) [b]	1.00 (Ref)	1.44 (0.96–2.17)	1.82 (1.32–2.52)	3.74 (2.79–5.03)
PAF (%) [c]	-	1.6	8.1	51.3
65–89 years				
N of at-risk	4,073	2,296	4,089	13,426
N of cardiovascular death	101	79	209	1,144
Person-years	24,688	15,834	33,143	100,104
Crude rate [a]	4.1	5.0	6.3	11.4
Sex/Age-adjusted HR (95% CI)	1.00 (Ref)	0.88 (0.65–1.18)	1.09 (0.86–1.39)	1.58 (1.28–1.94)
Fully adjusted HR (95% CI) [b]	1.00 (Ref)	0.88 (0.65–1.18)	1.13 (0.89–1.43)	1.65 (1.34–2.04)
PAF (%) [c]	-	-0.7	1.5	29.5

[a] A crude rate was expressed as per 1,000 person-years.
[b] Covariates were sex, age, total cholesterol, current-/ex-smoking, current-/ex-drinking, body mass index (< 18.5/≥ 25 kg/m^2), and diabetes.
[c] PAF estimate was based on the HR obtained by the fully adjusted model.
[d] Hypertension included untreated participants with BP ≥ 140/≥ 90 mmHg and treated participants.
CI, confidence interval; HR, hazard ratio; BP, blood pressure; PAF, population-attributable fraction; NBP, normal BP; High-NBP, high-normal BP; Elev-BP, elevated BP; HT, hypertension

Supplementary Table 5. Leave-one-out analysis on the association between CVD risk and BP categories in untreated participants

	Hazard ratios (95% confidence intervals) for total CVD					
Excluded cohort (baseline year)	NBP	High-NBP	Elev-BP	GI-HT	GII-HT	GIII-HT
All [No cohort omitted]	1.00 (Ref)	1.12 (0.88–1.42)	1.43 (1.18–1.74)	1.92 (1.60–2.32)	2.36 (1.91–2.90)	3.03 (2.36–3.89)
"Ohsaki cohort (1995)" omitted	1.00 (Ref)	1.22 (0.94–1.58)	1.57 (1.27–1.95)	2.08 (1.69–2.56)	2.57 (2.05–3.22)	3.33 (2.56–4.34)
"Ohasama cohort (1987)" omitted	1.00 (Ref)	1.10 (0.87–1.41)	1.42 (1.17–1.73)	1.91 (1.58–2.31)	2.32 (1.88–2.86)	2.97 (2.30–3.82)
"YKK workers cohort (1990)" omitted	1.00 (Ref)	1.11 (0.87–1.41)	1.41 (1.16–1.72)	1.88 (1.55–2.27)	2.30 (1.86–2.84)	2.97 (2.31–3.82)
"RERF cohort (1986)" omitted	1.00 (Ref)	1.13 (0.88–1.46)	1.47 (1.19–1.81)	1.92 (1.57–2.34)	2.41 (1.93–3.02)	3.04 (2.31–4.00)
"Hisayama cohort (1988)" omitted	1.00 (Ref)	1.03 (0.80–1.33)	1.39 (1.13–1.70)	1.87 (1.54–2.27)	2.26 (1.82–2.81)	2.87 (2.20–3.73)
"NIPPON DATA80 (1980)" omitted	1.00 (Ref)	1.09 (0.82–1.45)	1.38 (1.10–1.74)	2.03 (1.63–2.52)	2.48 (1.92–3.19)	3.13 (2.27–4.32)
"NIPPON DATA90 (1990)" omitted	1.00 (Ref)	1.18 (0.92–1.51)	1.44 (1.17–1.76)	1.91 (1.57–2.33)	2.36 (1.89–2.95)	3.39 (2.61–4.40)
"JMS cohort (1994)" omitted	1.00 (Ref)	1.13 (0.87–1.47)	1.40 (1.13–1.73)	1.92 (1.57–2.36)	2.40 (1.91–3.00)	2.80 (2.14–3.67)
"Aichi workers cohort (2002)" omitted	1.00 (Ref)	1.12 (0.88–1.43)	1.44 (1.18–1.75)	1.93 (1.60–2.33)	2.35 (1.91–2.91)	3.04 (2.37–3.92)
"Iwate-Kenpoku cohort (2002)" omitted	1.00 (Ref)	1.06 (0.82–1.36)	1.39 (1.14–1.69)	1.86 (1.54–2.25)	2.20 (1.78–2.73)	2.90 (2.25–3.75)

BP, blood pressure; NBP, normal BP; High-NBP, high-normal BP; Elev-BP, elevated BP; GI/GII/GIII-HT, grade I/II/III hypertension; YKK, Yoshikawa Kogyo Kabushiki Kaisha; RERF, the Radiation Effects Research Foundation; NIPPON DATA80/90, National Integrated Project for Prospective Observation of Non-communicable Disease and its Trends in the Aged 1980/1990; JMS, the Jichi Medical School

Supplementary Table 6. Leave-one-out analysis on the association between CVD risk and hypertension

	Hazard ratios (95% confidence intervals) for total CVD			
Excluded cohort (baseline year)	NBP	High-NBP	Elev-BP	HT [BP ≥140/≥90 mmHg or treated]
All [No cohort omitted]	1.00 (Ref)	1.10 (0.86–1.40)	1.39 (1.15–1.68)	2.29 (1.93–2.72)
"Ohsaki cohort (1995)" omitted	1.00 (Ref)	1.19 (0.92–1.55)	1.52 (1.23–1.89)	2.44 (2.01–2.96)
"Ohasama cohort (1987)" omitted	1.00 (Ref)	1.09 (0.86–1.39)	1.38 (1.14–1.68)	2.27 (1.91–2.70)
"YKK workers cohort (1990)" omitted	1.00 (Ref)	1.10 (0.86–1.40)	1.37 (1.13–1.67)	2.26 (1.90–2.68)
"RERF cohort (1986)" omitted	1.00 (Ref)	1.11 (0.86–1.43)	1.42 (1.15–1.74)	2.31 (1.93–2.78)
"Hisayama cohort (1988)" omitted	1.00 (Ref)	1.01 (0.79–1.31)	1.35 (1.10–1.64)	2.22 (1.86–2.65)
"NIPPON DATA80 (1980)" omitted	1.00 (Ref)	1.07 (0.81–1.43)	1.35 (1.08–1.69)	2.33 (1.92–2.84)
"NIPPON DATA90 (1990)" omitted	1.00 (Ref)	1.16 (0.90–1.48)	1.39 (1.14–1.70)	2.28 (1.90–2.73)
"JMS cohort (1994)" omitted	1.00 (Ref)	1.13 (0.87–1.46)	1.37 (1.11–1.69)	2.30 (1.91–2.79)
"Aichi workers cohort (2002)" omitted	1.00 (Ref)	1.11 (0.87–1.41)	1.40 (1.15–1.70)	2.29 (1.93–2.73)
"Iwate-Kenpoku cohort (2002)" omitted	1.00 (Ref)	1.05 (0.82–1.34)	1.36 (1.12–1.65)	2.23 (1.86–2.66)

BP, blood pressure; NBP, normal BP; High-NBP, high-normal BP; Elev-BP, elevated BP; HT, hypertension; YKK, Yoshikawa Kogyo Kabushiki Kaisha; RERF, the Radiation Effects Research Foundation; NIPPON DATA80/90, National Integrated Project for Prospective Observation of Non-communicable Disease and its Trends in the Aged 1980/1990; JMS, the Jichi Medical School

Supplementary Table 7. Cardiovascular mortality by subtype according to BP categories in untreated participants

Event type<br>Data in each age group	NBP	High-NBP	Elev-BP	GI-HT	GII-HT	GIII-HT
N	18,355	7,526	15,442	11,772	3,592	969
Person-years	164,078	74,551	173,252	129,565	41,160	11,645
**Total stroke**, N of deaths	67	47	134	236	127	53
Crude rate [a]	0.4	0.6	0.8	1.8	3.1	4.6
Sex/Age-adjusted HR (95% CI)	1.00 (Ref)	1.00 (0.69–1.45)	1.24 (0.92–1.67)	1.89 (1.43–2.49)	2.49 (1.83–3.38)	3.03 (2.09–4.40)
Fully adjusted HR (95% CI) [b]	1.00 (Ref)	1.00 (0.69–1.46)	1.27 (0.94–1.71)	1.93 (1.45–2.56)	2.54 (1.86–3.47)	3.13 (2.15–4.57)
PAF (%) [c]	-	0.0	4.3	17.1	11.6	5.4
**Ischemic stroke**, N of deaths	39	26	65	123	68	26
Crude rate [a]	0.2	0.3	0.4	0.9	1.7	2.2
Sex/Age-adjusted HR (95% CI)	1.00 (Ref)	0.82 (0.50–1.35)	0.89 (0.60–1.33)	1.32 (0.91–1.92)	1.60 (1.06–2.42)	1.79 (1.07–2.99)
Fully adjusted HR (95% CI) [b]	1.00 (Ref)	0.82 (0.50–1.35)	0.91 (0.61–1.36)	1.34 (0.92–1.95)	1.65 (1.09–2.50)	1.87 (1.11–3.15)
PAF (%) [c]	-	-1.7	-1.9	9.0	7.7	3.5
**ICH**, N of deaths	12	9	31	59	28	18
Crude rate [a]	0.1	0.1	0.2	0.5	0.7	1.5
Sex/Age-adjusted HR (95% CI)	1.00 (Ref)	1.23 (0.52–2.92)	1.84 (0.94–3.62)	3.32 (1.76–6.26)	4.22 (2.10–8.48)	8.13 (3.81–17.37)
Fully adjusted HR (95% CI) [b]	1.00 (Ref)	1.26 (0.53–3.00)	1.97 (1.00–3.88)	3.63 (1.91–6.89)	4.66 (2.30–9.42)	9.18 (4.26–19.80)
PAF (%) [c]	-	1.2	9.7	27.2	14.0	10.2
**CHD**, N of deaths	24	20	89	104	48	17
Crude rate [a]	0.1	0.3	0.5	0.8	1.2	1.5
Sex/Age-adjusted HR (95% CI)	1.00 (Ref)	1.19 (0.65–2.16)	2.13 (1.34–3.39)	2.25 (1.42–3.57)	2.56 (1.54–4.27)	2.68 (1.41–5.09)
Fully adjusted HR (95% CI) [b]	1.00 (Ref)	1.19 (0.65–2.17)	2.20 (1.38–3.51)	2.29 (1.44–3.64)	2.67 (1.59–4.47)	2.87 (1.50–5.49)
PAF (%) [c]	-	1.0	16.1	19.4	9.9	3.7
**Heart Failure**, N of deaths	30	24	46	93	48	22
Crude rate [a]	0.2	0.3	0.3	0.7	1.2	1.9
Sex/Age-adjusted HR (95% CI)	1.00 (Ref)	1.06 (0.61–1.81)	0.85 (0.53–1.35)	1.39 (0.91–2.13)	1.60 (0.99–2.58)	2.00 (1.13–3.55)
Fully adjusted HR (95% CI) [b]	1.00 (Ref)	1.09 (0.63–1.87)	0.92 (0.58–1.48)	1.58 (1.03–2.44)	1.87 (1.15–3.05)	2.47 (1.38–4.42)
PAF (%) [c]	-	0.7	-1.5	13.0	8.5	5.0

[a] A crude rate was expressed as per 1,000 person-years.

[b] Covariates were sex, age, total cholesterol, current-/ex-smoking, current-/ex-drinking, body mass index (< 18.5/≥ 25 kg/m^2), and diabetes.

[c] PAF estimate was based on the HR obtained by the fully adjusted model.

CI, confidence interval; HR, hazard ratio; BP, blood pressure; PAF, population-attributable fraction; NBP, normal BP; High-NBP, high-normal BP; Elev-BP, elevated BP; GI/GII/GIII-HT, grade I/II/III hypertension; ICH, intracerebral hemorrhage; CHD, coronary heart disease

Supplementary Table 8. Cardiovascular mortality by subtype when including treated participants in the HT group

Event type<br>Data in each age group	NBP	High-NBP	Elev-BP	HT [d]
N	18,355	7,526	15,442	29,247
Person-years	164,078	74,551	173,252	287,793
**Total stroke**, N of deaths	67	47	134	736
Crude rate [a]	0.4	0.6	0.8	2.6
Sex/Age-adjusted HR (95% CI)	1.00 (Ref)	0.99 (0.68–1.44)	1.21 (0.90–1.63)	2.27 (1.76–2.94)
Fully adjusted HR (95% CI) [b]	1.00 (Ref)	0.99 (0.68–1.44)	1.24 (0.92–1.66)	2.33 (1.79–3.02)
PAF (%) [c]	-	0.0	2.6	42.6
**Ischemic stroke**, N of deaths	39	26	65	389
Crude rate [a]	0.2	0.3	0.4	1.4
Sex/Age-adjusted HR (95% CI)	1.00 (Ref)	0.83 (0.50–1.36)	0.88 (0.59–1.32)	1.63 (1.16–2.29)
Fully adjusted HR (95% CI) [b]	1.00 (Ref)	0.82 (0.50–1.35)	0.90 (0.60–1.34)	1.65 (1.17–2.32)
PAF (%) [c]	-	-1.1	-1.4	29.5
**ICH**, N of deaths	12	9	31	168
Crude rate [a]	0.1	0.1	0.2	0.6
Sex/Age-adjusted HR (95% CI)	1.00 (Ref)	1.19 (0.50–2.84)	1.78 (0.91–3.49)	3.67 (2.02–6.68)
Fully adjusted HR (95% CI) [b]	1.00 (Ref)	1.22 (0.51–2.90)	1.87 (0.96–3.67)	3.97 (2.17–7.26)
PAF (%) [c]	-	0.7	6.6	57.1
**CHD**, N of deaths	24	20	89	307
Crude rate [a]	0.1	0.3	0.5	1.1
Sex/Age-adjusted HR (95% CI)	1.00 (Ref)	1.15 (0.64–2.09)	2.08 (1.32–3.27)	2.53 (1.66–3.86)
Fully adjusted HR (95% CI) [b]	1.00 (Ref)	1.15 (0.63–2.08)	2.12 (1.34–3.34)	2.53 (1.65–3.89)
PAF (%) [c]	-	0.6	10.7	42.2
**Heart Failure**, N of deaths	30	24	46	288
Crude rate [a]	0.2	0.3	0.3	1.0
Sex/Age-adjusted HR (95% CI)	1.00 (Ref)	1.04 (0.60–1.78)	0.82 (0.52–1.31)	1.60 (1.08–2.36)
Fully adjusted HR (95% CI) [b]	1.00 (Ref)	1.04 (0.61–1.79)	0.87 (0.55–1.39)	1.77 (1.19–2.63)
PAF (%) [c]	-	0.2	-1.8	32.2

[a] A crude rate was expressed as per 1,000 person-years.
[b] Covariates were sex, age, total cholesterol, current-/ex-smoking, current-/ex-drinking, body mass index (< 18.5/≥ 25 kg/m^2), and diabetes.
[c] PAF estimate was based on the HR obtained by the fully adjusted model.
[d] Hypertension included untreated participants with BP ≥ 140/≥ 90 mmHg and treated participants.
CI, confidence interval; HR, hazard ratio; BP, blood pressure; PAF, population-attributable fraction; NBP, normal BP; High-NBP, high-normal BP; Elev-BP, elevated BP; HT, hypertension; ICH, intracerebral hemorrhage; CHD, coronary heart disease

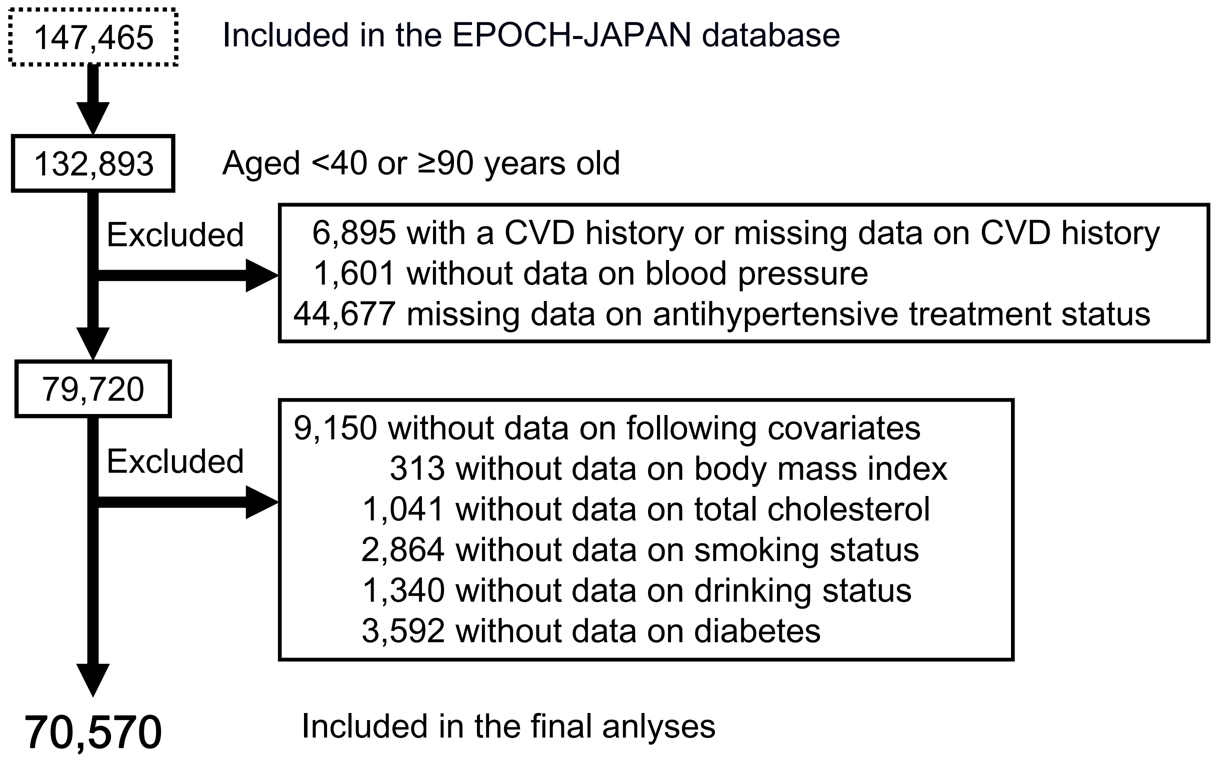

Supplementary Figure 1. Participants' selection flow

After applying all criteria, we extracted 70,570 participants from 10 cohorts (Ohsaki, Ohasama, Yoshikawa Kogyo Kabushiki Kaisha [YKK] Workers', the Radiation Effects Research Foundation [RERF], Hisayama Study, National Integrated Project for Prospective Observation of Non-communicable Disease and its Trends in the Aged 1980 [NIPPON DATA80], NIPPON DATA90, the Jichi Medical School [JMS], Aichi Workers,' and Iwate-Kenpoku Cohort Studies).

EPOCH-JAPAN, the Evidence for Cardiovascular Prevention from Observational Cohorts in Japan; CVD, cardiovascular disease

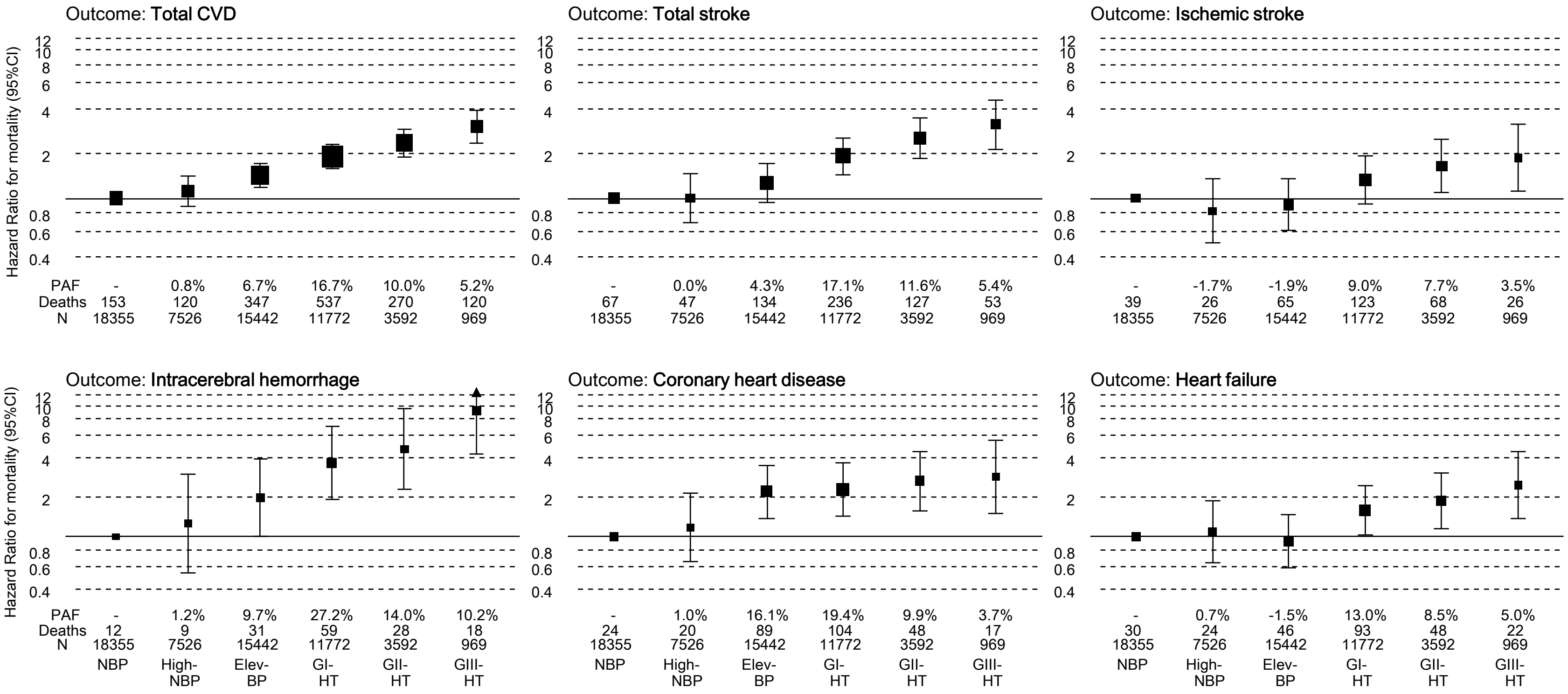

Supplementary Figure 2. Cardiovascular mortality by subtype according to BP categories in untreated participants

Detailed data are represented in **Supplementary Table 3** for total CVD deaths and **Supplementary Table 5** for deaths from other CVD subtypes**.** Hazard ratios were adjusted by sex, age, total cholesterol, ex-smoking, current smoking, ex-drinking, current drinking, body mass index (BMI, < 18.5, and ≥ 25 kg/m^2), and diabetes. The sizes of the marker boxes indicate the number of events in each group. BP, blood pressure; NBP, normal BP; High-NBP, high-normal BP; Elev-BP, elevated BP; GI/GII/GIII-HT, grade I/II/III hypertension; CVD, cardiovascular disease

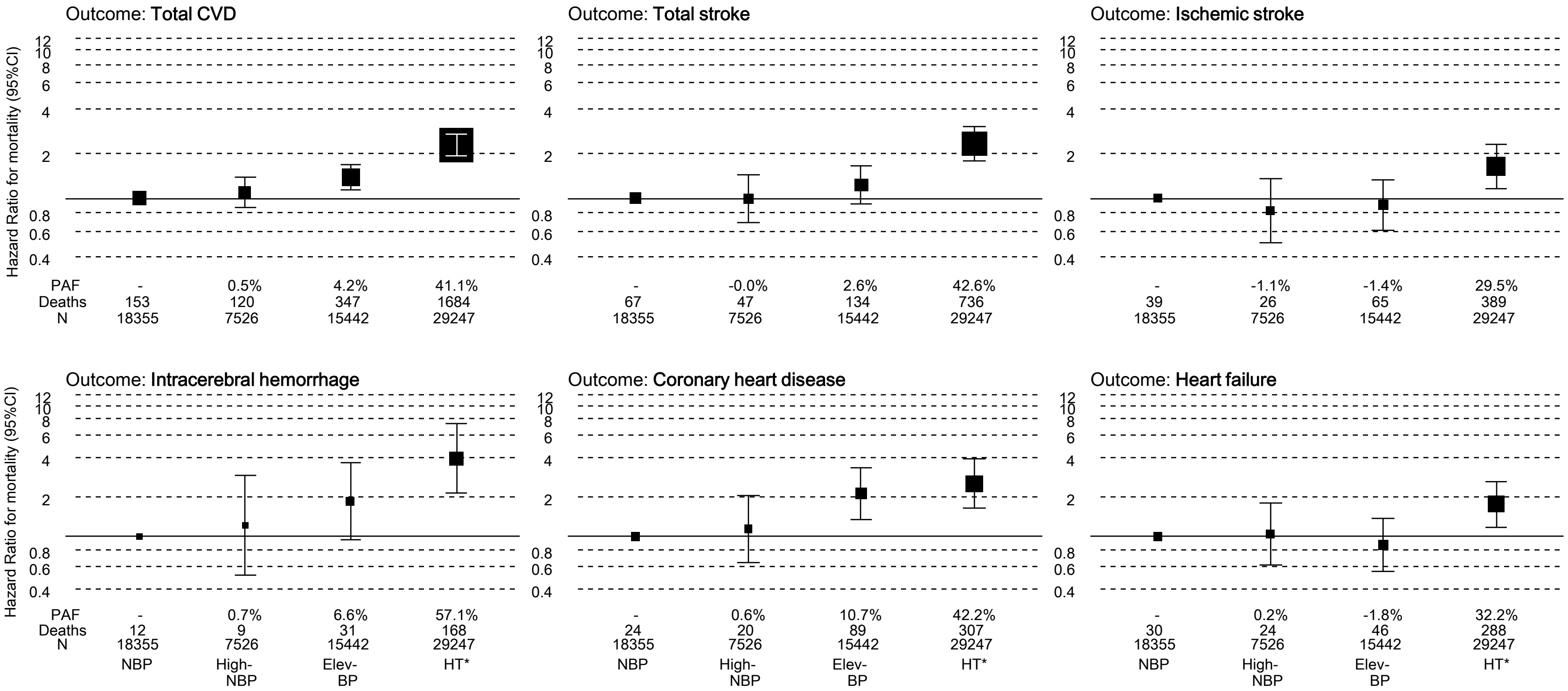

Supplementary Figure 3. Cardiovascular mortality by subtype according to BP categories when including treated participants in the HT group

Detailed data are shown in **Supplementary Table 4** for total CVD deaths and **Supplementary Table 6** for deaths from other CVD subtypes**.** The hazard ratios were adjusted by sex, age, total cholesterol, ex-smoking, current smoking, ex-drinking, current drinking, body mass index (< 18.5 and ≥ 25 kg/m^2), and diabetes. The sizes of the marker boxes indicate the number of events in each group.
BP, blood pressure; NBP, normal BP; High-NBP, high-normal BP; Elev-BP, elevated BP; HT, hypertension (*untreated participants with BP ≥ 140/≥ 90 mmHg and treated participants)
